# Phase-Dependent Shifting of the Adrenal Clock by Acute Stress-Induced ACTH

**DOI:** 10.3389/fendo.2016.00081

**Published:** 2016-06-29

**Authors:** William C. Engeland, J. Marina Yoder, Carley A. Karsten, Paulo Kofuji

**Affiliations:** ^1^Department of Neuroscience, University of Minnesota, Minneapolis, MN, USA

**Keywords:** adrenal clock, acute stress, restraint stress, dexamethasone, ACTH, circadian, mPER2:LUC

## Abstract

The adrenal cortex has a molecular clock that generates circadian rhythms in glucocorticoid production, yet it is unclear how the clock responds to acute stress. We hypothesized that stress-induced ACTH provides a signal that phase shifts the adrenal clock. To assess whether acute stress phase shifts the adrenal clock *in vivo* in a phase-dependent manner, mPER2:LUC mice on a 12:12-h light:dark cycle underwent restraint stress for 15 min or no stress at zeitgeber time (ZT) 2 (early subjective day) or at ZT16 (early subjective night). Adrenal explants from mice stressed at ZT2 showed mPER2:LUC rhythms that were phase-advanced by ~2 h, whereas adrenals from mice stressed at ZT16 showed rhythms that were phase-delayed by ~2 h. The biphasic response was also observed in mice injected subcutaneously either with saline or with ACTH at ZT2 or ZT16. Blockade of the ACTH response with the glucocorticoid, dexamethasone, prevented restraint stress-induced phase shifts in the mPER2:LUC rhythm both at ZT2 and at ZT16. The finding that acute stress results in a phase-dependent shift in the adrenal mPER2:LUC rhythm that can be blocked by dexamethasone indicates that stress-induced effectors, including ACTH, act to phase shift the adrenal clock rhythm.

## Introduction

The suprachiasmatic nucleus (SCN) is responsible for generating circadian rhythms in mammals (1). The molecular clock that underlies SCN rhythmicity is found in most mammalian cells (2), providing a peripheral clock mechanism that subserves tissue-specific functional rhythms (3). A fundamental question is how peripheral clocks are entrained to light and to other environmental signals (4). The adrenal cortex expresses a clock that can control the corticosterone rhythm by inducing rhythmic expression of the clock-controlled gene, steroidogenic acute regulatory (StAR) protein (5). Knockdown of the adrenal clock results in a corticosterone rhythm with reduced amplitude under constant dark, yet the rhythm is maintained under light–dark (LD) conditions (5). These results argue for redundancy in controlling corticosterone rhythms, with both clock-dependent and clock-independent mechanisms.

Our results using the mPER2:LUC mouse have shown that the adrenal clock can be reset *in vitro* by ACTH in a phase-dependent manner (6). The *in vitro* experiments showed that ACTH produced a phase delay when administered at a circadian time analogous to subjective night, but no phase shift at the circadian time representing subjective day. To examine whether stress is capable of phase-shifting the adrenal clock *in vivo*, we examined the adrenal clock in mPER2:LUC mice after exposure to chronic subordinate stress (7). The chronic subordinate stress model consisted of a brief period (10 min or less) of daily physical contact between subordinate and dominant mice at early subjective day [zeitgeber time (ZT) 3] followed by cohabitation for the remainder of the 24-h period. We found that a single exposure to subordination stress produced a phase advance in the adrenal mPER2:LUC rhythm that was maintained after 14 days of chronic subordinate stress. However, it is unclear (1) whether the effect of acute stress on the adrenal clock is dependent on the phase of the circadian rhythm and (2) whether the phase shift depends on the release of ACTH. Using acute stress to activate the hypothalamic–pituitary–adrenal (HPA) axis in mPER2:LUC mice, experiments were done to determine whether stress shifts the phase of the adrenal clock in a phase-dependent fashion and whether the adrenal response occurs after blockade of ACTH release.

## Materials and Methods

### Animals

Homozygous male mPER2:LUC mice (8) (3–6 months old) bred in-house were housed on a 12:12-h LD cycle (lights on at 0600 hours). Animals were maintained and cared for in accordance with the NIH Guide for the Care and Use of Laboratory Animals. Experimental procedures were approved by the University of Minnesota Animal Care and Use Committee.

### Bioluminescence

Animals were killed by decapitation 3.5–4.0 h before lights out (at ZT 8–8.5). Adrenals were rapidly excised and placed in cold Hank’s balanced salt solution. Cleaned and hemisected adrenals were placed on Millicell organotypic inserts (PICMORG50, 30 mm hydrophilic PTFE membrane, 0.4 μm pore size) in a 35 mm Petri dish with 1.5 ml of warmed culture media (Dulbecco’s Modified Eagle media w/o Phenol Red) supplemented with luciferin and penicillin/streptomycin, as described previously (6, 8). Dishes were sealed with circular glass coverslips and silicon grease. Cultures were maintained at 36°C, and bioluminescence was measured using photomultiplier tubes in an Actimetrics Lumicycle.

### Experiments

#### Experiment 1a

Male mPER2:LUC mice (*n* = 7/group) housed under a 12:12 L:D cycle underwent a 15-min restraint stress or no stress at ZT2. Mice were restrained by being placed in 50 ml conical tubes (Fisher Scientific, cat. # 05-539-13) with air holes drilled in tube bottoms to permit respiration. Mice were returned to their home cages following restraint and killed by decapitation at ZT8. Adrenals were processed for monitoring rhythms in bioluminescence.

#### Experiment 1b

Male mPER2:LUC mice (*n* = 6/group) housed under a 12:12 L:D cycle underwent a 15-min restraint stress or no stress at ZT16; at ZT16 all procedures were performed under dim red light. Mice were returned to their home cages following restraint and killed by decapitation on the next day at ZT8. Adrenals were processed for monitoring rhythms in bioluminescence.

#### Experiment 2a

Male mPER2:LUC mice (*n* = 5–6/group) housed under a 12:12 L:D cycle were injected with saline (100 μl sc; vehicle for ACTH) or ACTH (0.3 μg/100 μl sc) or underwent no stress at ZT2. Mice were returned to their home cages following injection and killed by decapitation at ZT8. Adrenals were processed for monitoring rhythms in bioluminescence.

#### Experiment 2b

Male mPER2:LUC mice (*n* = 5/group) housed under a 12:12 L:D cycle were injected with saline (100 μl sc; vehicle for ACTH) or ACTH (0.3 μg/100 μl sc) or no stress at ZT16 under dim red light. Mice were returned to their home cages following restraint and killed by decapitation on the next day at ZT8. Adrenals were processed for monitoring rhythms in bioluminescence.

#### Experiment 3

Male mPER2:LUC mice (*n* = 4–5/group) were pretreated with dexamethasone [Dexamethasone phosphate (Bimeda Inc.); 250 μg/kg BW sc] or needle puncture only as a control at ZT14 and underwent restraint stress for 15 min or no stress at ZT16. Mice were decapitated immediately following restraint, and trunk blood was collected for hormone assay. Plasma ACTH and corticosterone were measured by RIA, as described previously (9).

#### Experiment 4a

Male mPER2:LUC mice (*n* = 4–5/group) were pretreated with dexamethasone (250 μg/kg BW sc) or needle puncture at ZT1 and underwent restraint stress for 15 min or no stress at ZT3. For experiment 4, the dexamethasone treatment was given at ZT1, instead of ZT0, to avoid manipulation of mice at light onset. To maintain a 2-h exposure to dexamethasone before stress, acute restraint was initiated at ZT3. Mice were returned to their home cages following restraint and killed by decapitation at ZT8. Adrenals were processed for monitoring rhythms in bioluminescence.

#### Experiment 4b

Male mPER2:LUC mice (*n* = 4–5/group) were pretreated with dexamethasone (250 μg/kg BW sc) or needle puncture at ZT14 and underwent restraint stress for 15 min or no stress at ZT16. Mice were returned to their home cages following restraint and killed by decapitation the next day at ZT8. Adrenals were processed for monitoring rhythms in bioluminescence.

### Data Analysis

Data from the first day of recording were omitted from analysis due to transient bioluminescent activity (10). The remaining data were smoothed and detrended using a 2- and 24-h running average, baseline subtracted, and fit to a damped sine wave using Lumicycle Analysis software (Actimetrics). Only tissue showing rhythms with a goodness of fit >85% were accepted. To assess changes in the adrenal clock rhythm induced by *in vivo* manipulations, phase was determined from the peak measured on the second day of incubation, and period was calculated using data from two cycles spanning the second and third days of incubation.

### Statistical Analysis

Data are presented as means ± SEM. Statistical differences were determined using one-way ANOVA (using Dunnett’s correction for *post hoc* analysis), two-way ANOVA (using Bonferroni’s correction for *post hoc* analysis), or unpaired Student’s *t*-test, where appropriate, using Prism software (GraphPad). Differences were considered significant if *p* < 0.05.

## Results

**Figure 1:**
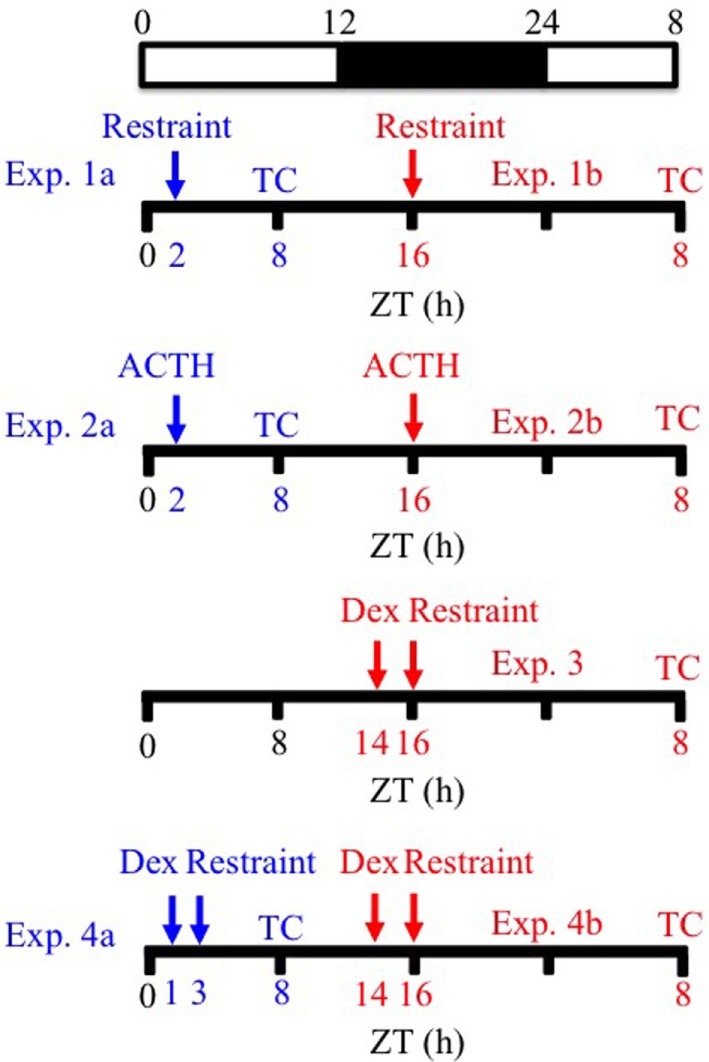
**Schematic showing the time-line for each experiment relative to zeitgeber time (ZT)**. Mice were on a 12:12-h LD cycle (0600:1800 hours) with lights on at ZT0 and lights off at ZT12. Experimental manipulations were done in early subjective day (denoted by blue font) or early subjective night (denoted by red font). Dex, dexamethasone; TC, time of tissue collection.

**Figure 2:**
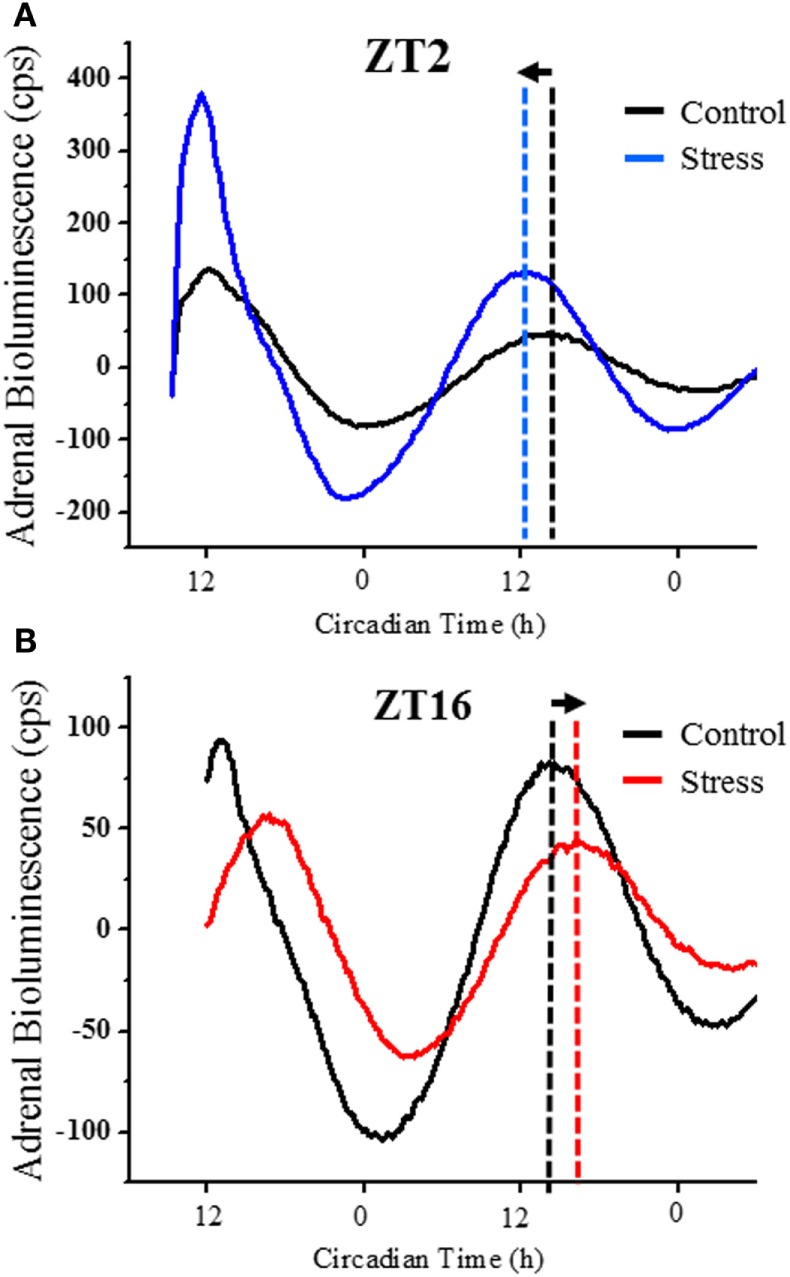
**Representative experiment showing the mPER2:LUC rhythm after detrending and baseline subtraction; adrenal explants were collected from control mice (black line) and mice exposed to acute (15 min) restraint stress at ZT2 (blue line) (A) or at ZT16 (red line) (B)**. The peak in adrenal PER2 bioluminescence is phase-advanced by ~2 h following restraint stress at ZT2 **(A)** and phase-delayed by ~2 h following restraint stress at ZT16 **(B)**. The vertical dashed lines identify the time of the peak phase in the mPER2:LUC rhythm. Adrenal bioluminescence is reported as counts per second (cps).

**Figure 3:**
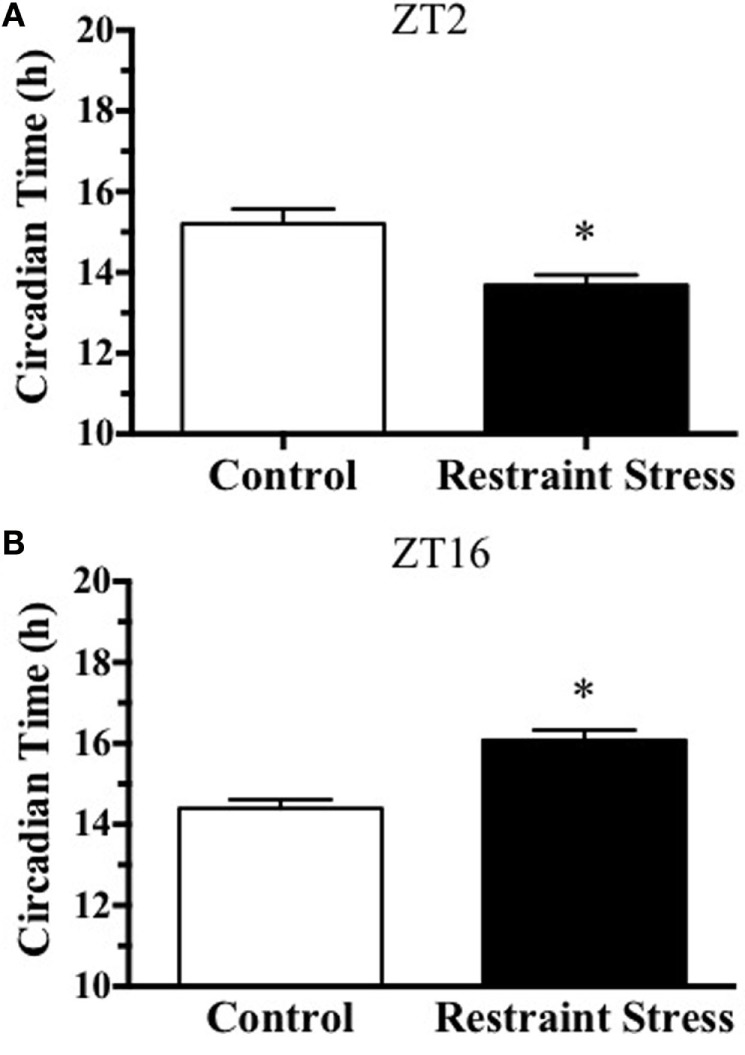
**Effect of acute (15 min) restraint stress at ZT2 (A) and at ZT16 (B) on the peak phase of the *in vitro* adrenal mPER2:LUC rhythm**. Values represent means ± SEM (*n* = 6–7/group). Based on unpaired Student’s *t*-test, *p < 0.05 vs. Control at ZT2 and ZT16.

A schematic showing the time line of treatments for each experiment as shown in Figure 1. Experiment 1 was done to determine if acute restraint stress results in a phase-dependent shift in the adrenal clock rhythm. Representative examples of mPER2:LUC bioluminescent activity are shown for adrenals collected from non-stressed mice and mice exposed to a 15-min restraint stress at ZT2 (Figure 2A) or at ZT16 (Figure 2B). Adrenals from mice that underwent a 15-min restraint stress at ZT2 showed a phase advance in the mPER2:LUC rhythm compared with adrenals from non-stressed (control) mice (Figure 3A), whereas adrenal rhythms from mice that were stressed at ZT16 showed a phase delay compared with controls (Figure 3B). Acute restraint stress had no effect on the period of the rhythm at ZT2 (CTRL: 23.25 ± 0.11; RESTRAINT: 23.32 ± 0.23) or at ZT16 (CTRL: 23.37 ± 0.08; RESTRAINT: 22.78 ± 0.23).

**Figure 4:**
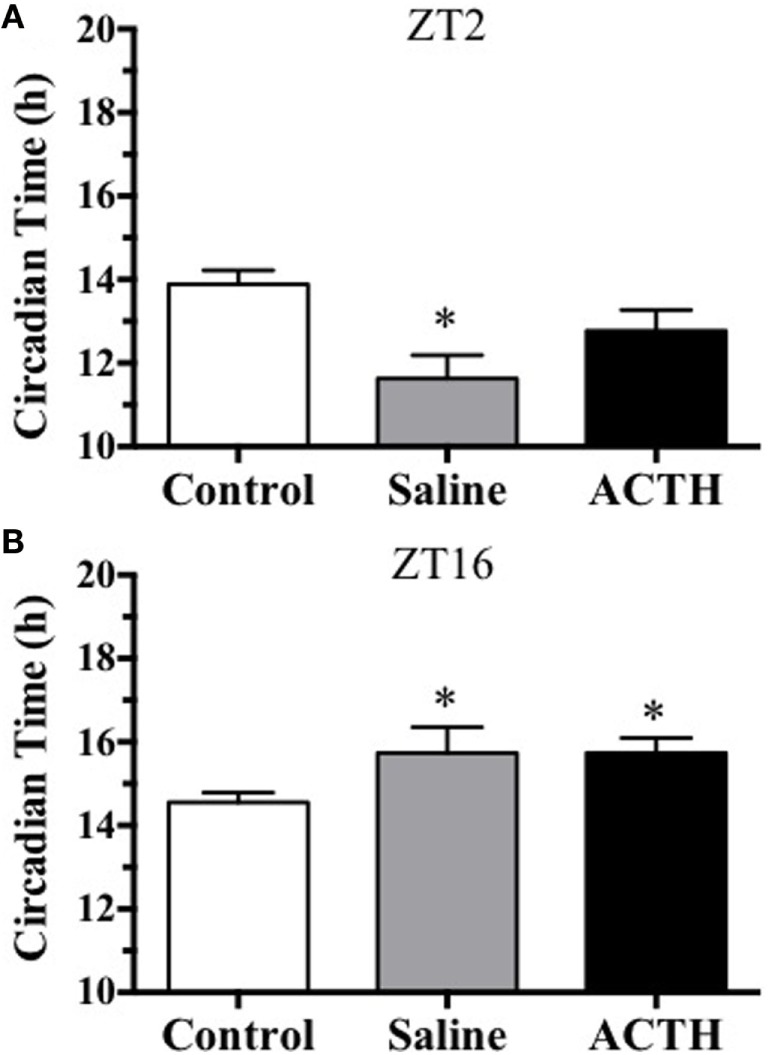
**Effect of injection of ACTH or saline or no treatment (Control) at ZT2 (A) and at ZT16 (B) on the peak phase of the *in vitro* adrenal mPER2:LUC rhythm**. Values represent means ± SEM (*n* = 5–6/group). Based on one-way ANOVA (using Dunnett’s correction for *post hoc* analysis), *p < 0.05 vs. Control at ZT2 and ZT16.

Two approaches were used to examine whether stress-induced ACTH contributed to stress-induced phase-dependent shifts in the adrenal mPER2:LUC rhythm. In experiment 2, mice were injected with a supramaximal dose of ACTH [100 μl, 3.0 μg/kg BW; Ref. (9)] and compared with mice injected with saline (100 μl) or no injection (Control) at ZT2 or ZT16. Adrenals from mice injected with saline at ZT2 showed a phase advance in the mPER2:LUC rhythm compared with adrenals from Control mice (Figure 4A), whereas rhythms from adrenals from mice injected with saline or ACTH at ZT16 showed a phase delay compared with Control mice (Figure 4B). No differences were observed between saline and ACTH at ZT2 or ZT16, indicating that the handling and injection stress was sufficient to shift the adrenal mPER2:LUC rhythm in a phase-dependent fashion. Neither injection of saline nor ACTH had an effect on the period of the rhythm at ZT2 (CTRL: 23.60 ± 0.18; saline: 23.58 ± 0.23; ACTH: 22.88 ± 0.49) or at ZT16 (CTRL: 22.12 ± 0.10; saline: 22.48 ± 0.28; ACTH: 22.32 ± 0.25).

**Figure 5:**
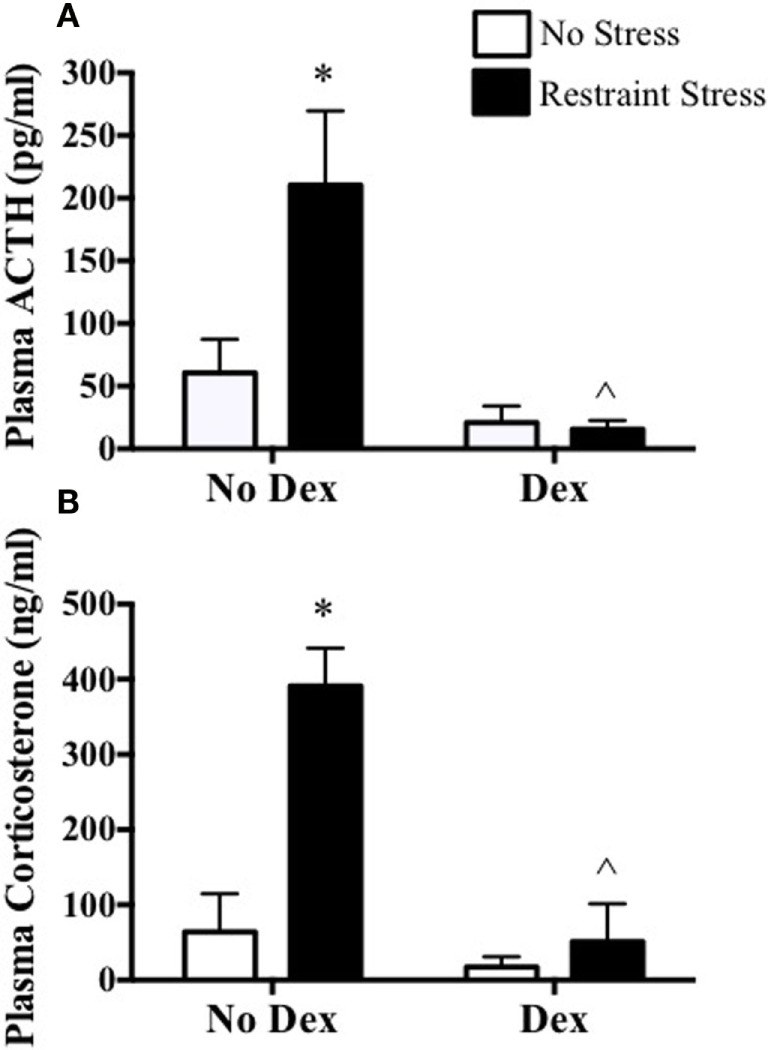
**Effect of dexamethasone blockade on restraint stress-induced plasma ACTH (A) and corticosterone (B) at ZT16 in mPER2:LUC mice**. Values represent means ± SEM (*n* = 4–5/group). Based on two-way ANOVA (using Bonferroni’s correction for *post hoc* analysis), *p < 0.05 vs. No Dex-No Stress and ^∧^*p* < 0.05 vs. No Dex-Stress.

**Figure 6:**
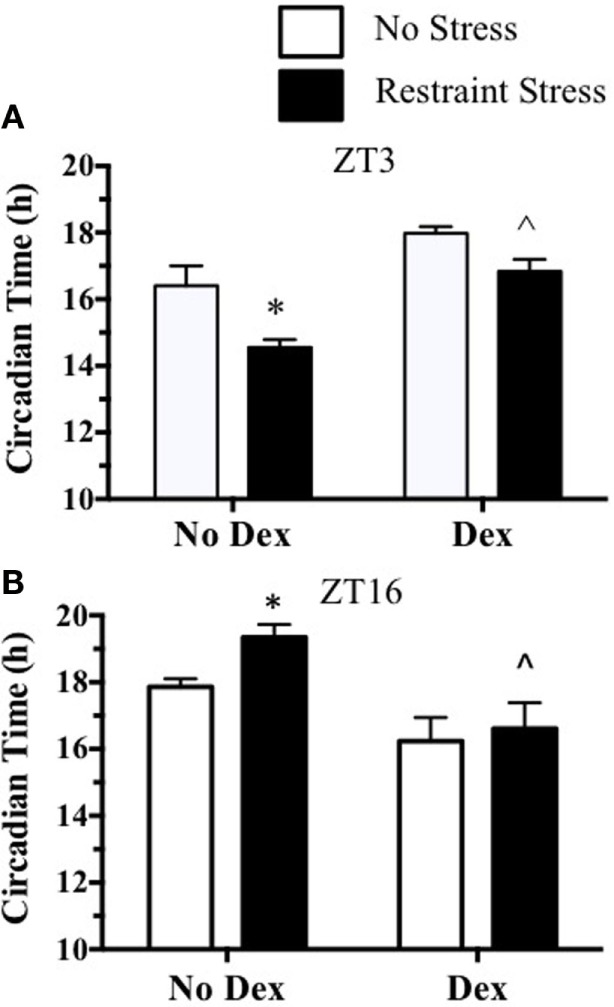
**Effect of dexamethasone treatment before restraint stress at ZT3 (A) or at ZT16 (B) on the peak phase of the *in vitro* adrenal mPER2:LUC rhythm**. Values represent means ± SEM (*n* = 4–5/group). Based on two-way ANOVA (using Bonferroni’s correction for *post hoc* analysis), *p < 0.05 vs. No Dex-No Stress and ^∧^*p* < 0.05 vs. No Dex-Stress at ZT3 and ZT16.

**Figure 7:**
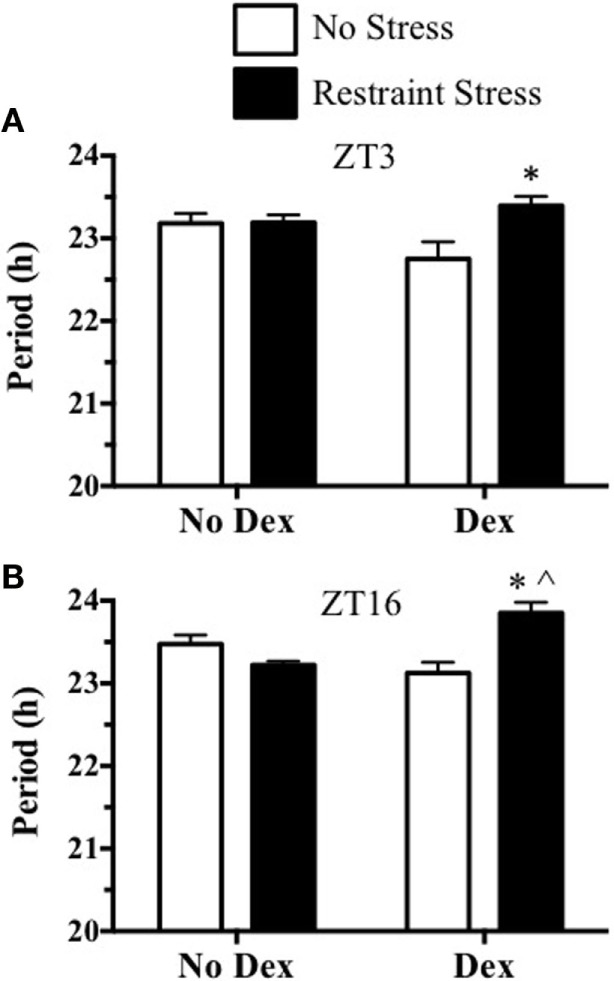
**Effect of dexamethasone treatment before restraint stress at ZT3 (A) or at ZT16 (B) on the period of the *in vitro* adrenal mPER2:LUC rhythm**. Values represent means ± SEM (*n* = 4–5/group). Based on two-way ANOVA (using Bonferroni’s correction for *post hoc* analysis), *p < 0.05 vs. Dex-No Stress and ^∧^*p* < 0.05 vs. No Dex-Stress at ZT3 and ZT16.

To further examine a possible role for stress-induced ACTH in the phase-dependent shift of the mPER2:LUC rhythm, dexamethasone was used to block ACTH release induced by restraint stress. In experiment 3, mice were pretreated with dexamethasone 2 h before undergoing restraint stress at ZT16. Plasma ACTH and corticosterone increased at 15 min after restraint in mice that were not treated with dexamethasone. In contrast, dexamethasone pretreatment blocked the plasma ACTH and corticosterone response to restraint (Figure 5). In experiment 4, dexamethasone was used to block ACTH 2 h prior to restraint at ZT3 or at ZT16, and adrenals were collected to measure changes in the mPER2:LUC rhythm *in vitro*. Results showed that dexamethasone prevented both the phase advance in the mPER2:LUC rhythm induced by restraint stress at ZT3 (Figure 6A) and the phase delay induced by restraint stress at ZT16 (Figure 6B), suggesting that increased ACTH induced by restraint stress contributes to the phase-dependent shift in the mPER2:LUC rhythm. Although the period of the mPER2:LUC rhythm was not affected by restraint stress in the absence of dexamethasone, the period was lengthened in adrenals from mice that underwent dexamethasone treatment followed by stress at ZT3 (Figure 7A) and ZT16 (Figure 7B).

## Discussion

Experiments were done to determine whether acute stress shifts the phase of the adrenal clock in a phase-dependent manner and whether the adrenal response is dependent on stress-induced release of ACTH. The present results show that a single 15-min restraint stress at ZT2 produced an ~2-h phase advance in the mPER2:LUC rhythm. In marked contrast, the same manipulation performed at ZT16 produced an ~2-h phase delay. To examine whether increased ACTH was responsible for the phase-dependent shift, we injected ACTH at ZT2 or at ZT16 to mimic restraint stress-induced ACTH. Injection stress was sufficient to phase shift the adrenal mPER2:LUC rhythm in a phase-dependent manner, yet responses to ACTH injection were comparable to saline injection. To assess a possible role for ACTH more clearly, mice were treated with dexamethasone to inhibit restraint stress-induced ACTH; results showed that dexamethasone blocked the phase-dependent shift in the mPER2:LUC rhythm. Taken together, these experiments show that acute stress is capable of producing a biphasic shift in the adrenal mPER2:LUC rhythm that can be blocked by dexamethasone, suggesting that ACTH is a necessary component in stress-induced alterations in adrenal clock timing.

Our previous work showed that a single exposure to subordination stress at early subjective day (ZT3) followed by cohabitation for the remainder of the day produced an ~2-h phase advance in the adrenal mPER2:LUC rhythm in male mice (7). By using a single acute restraint or injection stress, the present experiments were designed to allow more precise control of the timing of stress exposure. Similar to the response to single subordination stress, acute restraint or injection stress at early subjective day (ZT2–3) phase-advanced the adrenal mPER2:LUC rhythm. In addition, when acute stress was applied at early subjective night (ZT16), the adrenal mPER2:LUC rhythm was phase-delayed (Figures 3 and 4). Others have examined whether repeated stress results in a phase-dependent effect on the adrenal mPER2:LUC rhythm (11). A 2-h exposure to subordination stress in early subjective day for 3 weeks resulted in a phase advance in the adrenal rhythm, but repeated subordination stress in early subjective night did not produce a phase shift in the adrenal rhythm. The effect of repeated stress at early subjective day confirmed our previous work showing that the phase advance in the adrenal mPER2:LUC rhythm was maintained after chronic (14-day) subordination stress, indicating that changes in the timing of the adrenal clock did not habituate to chronic stress applied at ZT3 (7). Since exposure to a single acute stressor, but not repeated exposure to stress, results in a biphasic effect on the adrenal mPER2:LUC rhythm, it is possible that the phase delay produced by acute stress in early subjective night habituates to repeated exposure to stress. Additional experiments are required to test this possibility.

The observation that acute restraint stress shifts the phase of the adrenal mPER2:LUC rhythm led to experiments assessing whether ACTH contributed to the response. This possibility was based in part on our previous study showing that ACTH *in vitro* induced a phase shift of adrenal mPER2:LUC rhythm (6). The 2-h phase delay of the mPER2:LUC rhythm following acute restraint stress during the dark period (ZT16) parallels the phase-dependent delay produced by ACTH *in vitro*. In contrast, the phase advances in the mPER2:LUC rhythm observed after restraint stress during the light period (ZT2) was not mimicked by ACTH *in vitro* (6). By injecting ACTH *in vivo*, experiments were done to assess the effect of ACTH directly on the phase-dependent response. Results showed that ACTH injection was comparable to saline injection in changing the phase of the adrenal mPER2:LUC rhythm, suggesting that injection stress alone was sufficient for producing a phase-dependent shift (Figure 4). To examine whether ACTH responses to acute stress were required for the phase shift, mice were treated with dexamethasone, a synthetic glucocorticoid, to block ACTH secretion. This approach takes advantage of the known effect of dexamethasone to act at the anterior pituitary as a negative feedback signal to inhibit ACTH release (12). Pretreatment with a low dose (250 μg/kg) of dexamethasone, similar to the dose used to block stress-induced ACTH in rats (9, 13), effectively blocked both restraint stress-induced plasma ACTH and corticosterone (Figure 5). In addition, dexamethasone pretreatment prevented both the phase advance produced by restraint stress at ZT3 and the phase delay produced by restraint stress at ZT16 (Figure 6). These findings show that acute stress shifts the phase of the adrenal clock rhythm *in vivo* and suggest that the response results at least in part from increased plasma ACTH.

Activation of glucocorticoid receptors by dexamethasone can entrain other peripheral clocks (14, 15). Thus, in addition to ACTH blockade, it is possible that the activation of glucocorticoid receptors in the adrenal (16) could be involved in phase-dependent changes in the adrenal mPER2:LUC rhythm. Although the high doses (10 mg/kg) of dexamethasone result in phase-dependent shifts in the body temperature rhythm, lower doses like those used in the present study have no effect (17). By evaluating the effects of dexamethasone alone on the adrenal mPER2:LUC rhythm, we were unable to find differences in peak phases between non-stress groups treated with or without dexamethasone (Figure 6). However, whereas changes in the period were not observed after stress or dexamethasone alone, dexamethasone combined with stress at ZT3 and ZT16 prolonged the period of the mPER2:LUC rhythm (Figure 7). Additional experiments are necessary to determine whether the mechanism for period lengthening includes activation of adrenal glucocorticoid receptors.

In non-photic entrainment, SCN-controlled rhythms, such as locomotor activity, shift in response to acute arousal (18). Non-photic entrainment by arousal is more prevalent during subjective day (19) and is characterized by a phase advance (20). Since we observed a phase advance in the adrenal mPER2:LUC rhythm following stress at ZT2, it is possible that the adrenal response is dependent on a change in SCN activity. Although we did not assess SCN mPER2:LUC activity, others have shown that repeated subordination stress at ZT1–3 does not affect the phase of the SCN clock gene rhythm (11). Moreover, acute subordinate stress during subjective night in rats does not shift the phase of activity or temperature rhythms, reflections of SCN activity, suggesting that acute stress during the active period acts independently of the SCN (19, 21). Since mice were housed under a 12:12-h LD cycle in the present study, it is likely that photic entrainment predominates in maintaining the phase of the SCN clock over stress-related stimuli. The biphasic stress-induced phase shifts of the adrenal clock may not result from non-photic entrainment of the SCN but instead may represent a direct phase-dependent response of the adrenal clock to stress.

The mechanism for a phase-dependent response of the adrenal clock rhythm to stress is unclear. The ability to block the phase-resetting induced by stress with dexamethasone implicates ACTH. Since the magnitude of the ACTH response to stress differs diurnally (22), exposure to different levels of ACTH may contribute to phase dependency. However, our *in vitro* data showed that the phase response was not dependent on the magnitude of the ACTH stimulus, since high concentrations of ACTH administered at early CTs were ineffective in phase-shifting the mPER2:LUC rhythm (6). It is likely that factors, in addition to ACTH, play a role. Since increases in adrenal sympathetic activity synergize with ACTH to increase adrenal steroidogenic responses (23) and also increase adrenal Per expression (24), sympathetic neural input may be involved in altering timing of the adrenal clock. The adrenal cortex includes sympathetic innervation by vasoactive intestinal peptide (VIP)-ergic fibers (25). Corticosterone rhythms are dampened in constitutive VIP knockout (26, 27) and in VIP receptor knockout mice (28, 29), supporting the possibility that stress-induced VIPergic activity could complement ACTH in phase-shifting the adrenal clock. As shown for light-induced phase shifts of the SCN clock (30), phase-shifting of the adrenal clock may result from stress-induced increases in adrenal PER that vary with the time of stress to differentially change PER negative feedback on the molecular clock. Additional studies are required to assess this possibility.

The present results show that acute stress produces a shift in the adrenal clock gene rhythm that is phase-dependent. The physiological importance of this observation stems in large part from the role of the adrenal clock in regulating glucocorticoid secretion. Rhythms in plasma glucocorticoids are paralleled by rhythms in adrenal sensitivity to ACTH (9, 22, 31), and the adrenal clock is thought to control rhythmic steroidogenesis by gating sensitivity to ACTH (32). Since the adrenal clock controls circadian rhythms in glucocorticoid production (5), it will be important to determine whether acute stress also shifts the phase of the plasma glucocorticoid rhythm in a phase-dependent manner. Since glucocorticoid rhythms entrain other peripheral clocks (14, 33), altered glucocorticoid rhythms produced by stress-induced phase shifts could have effects on metabolic, hemodynamic, and cognitive function.

## Author Contributions

WE designed the experiments, assisted in performing the experiments, analyzed the results, wrote the initial draft of the manuscript, and completed the final edited version of the manuscript. JY and CK assisted in designing and performing the experiments, analyzing the results, and editing the manuscript. PK assisted in designing the experiments, performing the experiments, and editing the manuscript.

## Conflict of Interest Statement

The authors declare that the research was conducted in the absence of any commercial or financial relationships that could be construed as a potential conflict of interest.
